# Comparison of the new RIDA qLine Allergy multiparameter immunoblot and the ImmunoCAP Specific IgE test for the identification of clinically relevant food and aeroallergen allergies

**DOI:** 10.3389/falgy.2024.1496882

**Published:** 2025-01-10

**Authors:** Katharina K. Hahn, Marie C. Schuppe, Moritz M. Hollstein, Susann Forkel, Timo Buhl

**Affiliations:** Department of Dermatology, Venereology and Allergology, University Medical Centre Goettingen, Goettingen, Germany

**Keywords:** aeroallergen, food allergen, IgE testing, immunoblot, multiparameter

## Abstract

**Background:**

Multiparameter immunoblot testing is increasingly used as an alternative to multiple individual IgE analyses for type 1 allergies. This study investigated the performance of an inexpensive immunoblot method, the RIDA qLine allergy test system (R-Biopharm AG), vs. the current gold standard.

**Methods:**

Three study-specific panels with 57 individual allergens (food and aeroallergens) were analyzed in serum samples from 200 patients with signs and symptoms of IgE-mediated allergies, using both the RIDA qLine Allergy and the reference method, the ImmunoCAP Specific IgE test (Thermo Fisher Scientific). In case of divergent results, corresponding allergens were remeasured using the secondary reference method, the 3gAllergy Specific IgE Universal Kit (Siemens). The clinical diagnoses of the 200 patients were included. In addition, a cross-reactive carbohydrate determinant (CCD)-inhibitor was used in the testing to decrease the incidence of positive CCD bands.

**Results:**

The mean overall agreement of all food and aeroallergens with the reference methods was 94.9%. Qualitative evaluation showed an average negative percent agreement of 98.9% and an average positive percent agreement of 75.1% for all individual allergens after testing with both reference methods. The additional treatment of samples with the CCD inhibitor successfully reduced the occurrence of positive CCD reactivity after retesting.

**Conclusion:**

The comparative analysis of RIDA qLine Allergy with the reference methods for specific IgE detection revealed a strong correlation between serum IgE levels measured across these platforms and clinical presentations, while also highlighting the necessity for careful contextual interpretation of results. Standardized allergen extracts would improve independent comparisons of different allergy testing methods.

## 1 Introduction

Immunoglobulin E (IgE) plays a pivotal role in the pathophysiology of allergic diseases, acting as a key mediator in type 1 allergies. A comprehensive analysis of common allergens in Germany demonstrated in 2013 that 48.6% of the evaluated participants displayed allergen-specific IgE (sIgE) to at least one allergen in the serum (1). This contrasts with the lifetime prevalence of an allergic disease of 30.0%, which nevertheless has shown an increasing trend for IgE-mediated allergies over the last few decades (2). The detection of sIgE and the correlation with associated clinical symptoms are essential for an accurate diagnosis and the development of effective treatment approaches for allergic diseases. The presence of allergen-specific serum IgE indicates sensitization but must be verified against the clinical symptoms to confirm the diagnosis of a relevant allergy. Conversely, a negative result for allergen-specific IgE possesses a high negative predictive value for ruling out an IgE-mediated allergy (3). Skin prick tests are a primary tool in diagnostics due to their high sensitivity in identifying allergens and the instant availability of results. Measuring allergen-specific IgE is particularly advantageous for individuals with severe anaphylactic reactions, skin diseases at the prick test area, or those on certain medications preventing wheal formation, and is independent of the specialist's experience or the test site (4).

The specificity of IgE testing can be complicated by cross-reactivity, often due to IgE antibodies against cross-reactive carbohydrate determinants (CCDs), consisting of xylose and core 1,3-linked fucose residues on plant or insect glycoproteins (5, 6). The prevalence of CCD-reactive IgE induced by these complex carbohydrate structures, that are widespread on otherwise unrelated glycoproteins, has been shown to be as high as one-quarter of serum samples from allergic patients (7). The presence of anti-CCD IgE with low clinical relevance can result in false-positive outcomes via unspecific binding and has given rise to diverse CCD-blocking approaches to improve diagnostic accuracy (8, 9).

The radioallergosorbent test (RAST) was the first assay to detect allergen-specific IgE (10). It has been largely superseded by methods that offer quicker results and require less sample volume. The current gold standard method for quantitative single sIgE detection is the ImmunoCAP Specific IgE test (ThermoFisher), which is based on a solid-phase cellulose polymer coated with allergens to bind IgE from patient samples, enabling the detection even of low levels of sIgE (0.1 kUA/L) (11). Another commonly used method is the Immulite 3gAllergy Specific IgE Universal Kit (Siemens), which utilizes allergens that are covalently attached to biotinylated polylysine polymers in a liquid environment (12). In contrast to fluorescence-based measurements with the ImmunoCAP system, the 3gAllergy test system employs a chemiluminescence signal to determine IgE levels. Both technologies utilize single allergen extracts, and sIgE detection is facilitated by a secondary anti-IgE antibody, enabling quantitative measurements (13). Assays investigating single sIgE values to allergens are also referred to as monoplex assays.

**Figure 1:**
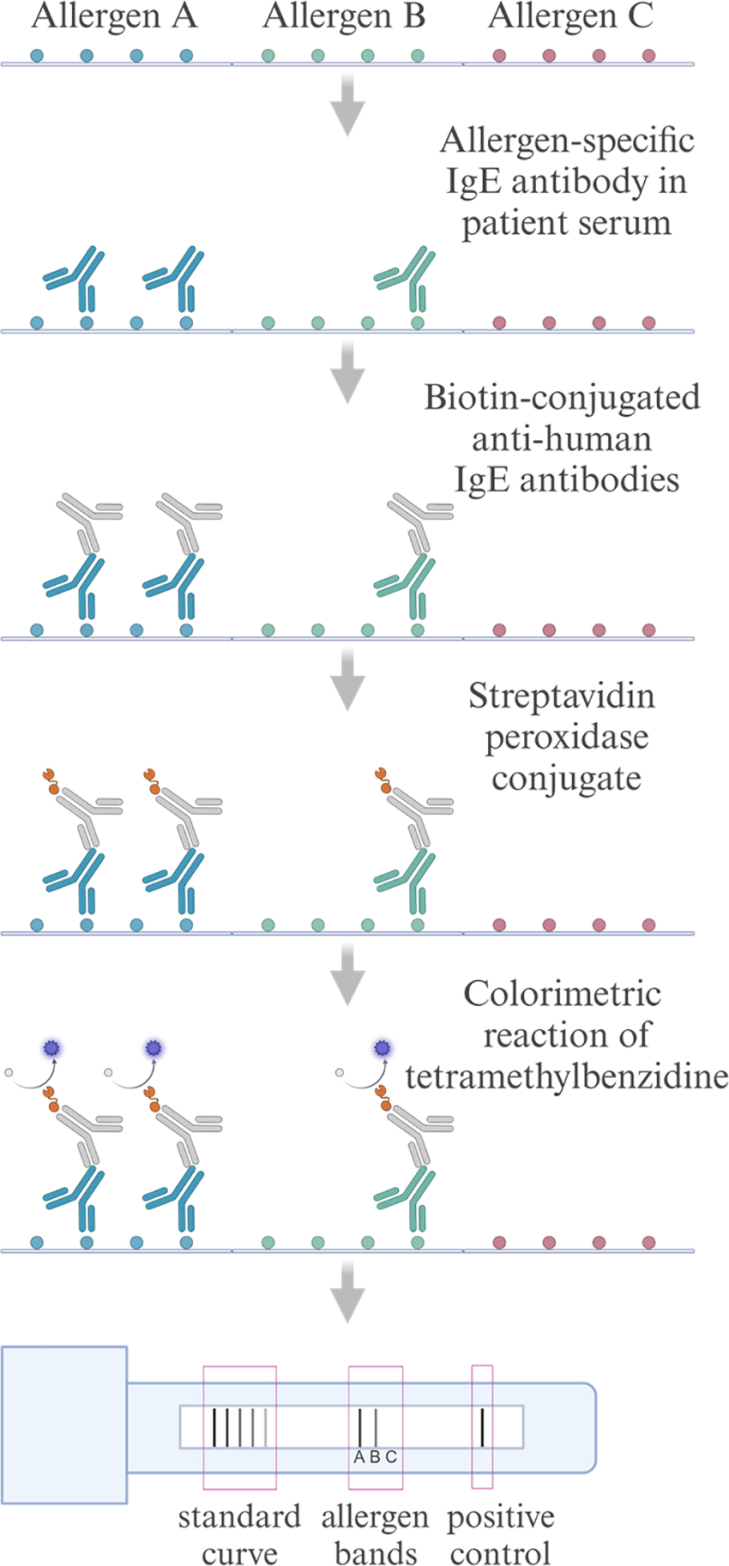
Schematic flowchart of the RIDA qLine Allergy test system for detecting allergen-specific IgE antibodies. Patient serum is applied to allergens immobilized on a nitrocellulose membrane, where allergen-specific IgE antibodies bind to their respective allergens. Biotin-conjugated anti-human IgE antibodies are subsequently added, followed by streptavidin-peroxidase conjugate, which binds to the biotin. The peroxidase catalyzes a reaction with the TMB substrate, producing a colorimetric change proportional to the concentration of a specific IgE. Color intensities are scanned and quantified against a standard curve. Created in https://BioRender.com.

To enhance cost-effectiveness and minimize sample volume requirements, multiplex technologies such as chip-based microarrays, bead-based immunoassays, and line blot assays, that enable the simultaneous analysis of multiple allergens, are becoming increasingly popular (14). The RIDA qLine Allergy test system (R-Biopharm) employs a manual or automated, multiparametric line immunoassay, where various allergen extracts are immobilized on a nitrocellulose membrane together with a standard curve present on the membrane, and the intensity of the color change from the substrate tetramethylbenzidine (TMB) is measured using a scanner (Figure 1). This setup facilitates qualitative and semi-quantitative detection of sIgE using minimal serum samples and operates independently of advanced laboratory instruments (15, 16).

This study was designed to evaluate the clinical performance of the RIDA qLine Allergy test system for the detection of sIgE in a cohort of patients with IgE-mediated allergies in comparison with the established gold standard ImmunoCAP system. In instances of discrepant results, comparisons were made using the 3gAllergy system. In addition, we compared the concordance of both laboratory tests with the clinical findings in our patient cohort and evaluated the improved clinical performance of the test after the introduction of the RIDA CCD inhibitor.

## 2 Patients and methods

### 2.1 Study design and study group

**Figure 2:**
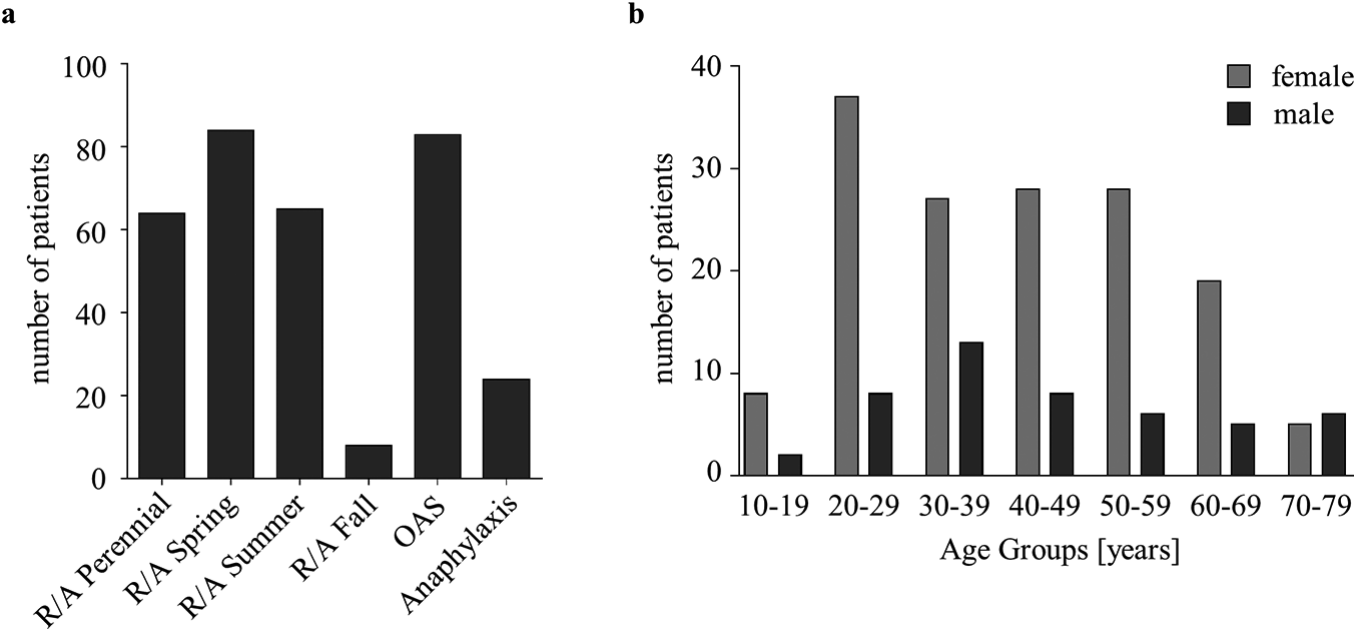
Basic characteristics of the study group. **(a)** Distribution of the 200 patients regarding the clinical diagnoses, categorized as follows: R/A: perennial (*n* = 64), spring season (*n* = 84), summer season (*n* = 65), and fall season (*n* = 8); OAS (*n* = 83); anaphylaxis (*n* = 24) to food allergens (depicted in total numbers; multiple mentions possible). **(b)** Distribution of sex and age in 10-year intervals, with the total number of patients depicted.

The study was designed as a single-center, observational, non-interventional, case–control study on anonymized and retrospectively collected residual human serum samples of patients with signs and symptoms of one or more IgE-mediated allergies as diagnosed by an allergist. After approval by the ethics committee of the University Medical Center Goettingen (ref. 01/12/21), 200 samples from routine diagnostics that were positive (sIgE ≥ 0.35 kU_A_/L; CAP class ≥ 1) for one or more allergens were included in the study. It was considered sufficient if 8–75 patients per tested allergen were CAP class ≥1, and at least 4 of these were CAP class ≥3 (as determined by the respective test method). As the number of positive sera for single, rare allergens was not achieved after analyzing 200 patients, the study samples were enriched for respective rare allergens using commercial samples with pre-analyzed positivity for these allergens. The 200 patient samples were tested for all 57 allergens and were characterized in more detail with information on sex, age (10-year intervals), and physician's suspected diagnosis before further serological diagnostics. The evaluation of the 200 patients by allergists comprised six categories of clinical diagnoses with multiple assignments possible. Patients with allergic rhinitis and/or allergic asthma (R/A) were categorized into R/A perennial (*n* = 64), spring season (*n* = 84), summer season (*n* = 65), and/or fall season (*n* = 8) (Figure 2a). Patients with food allergies were categorized as having oral allergy syndrome (OAS, *n* = 83) and/or food-induced anaphylaxis (*n* = 24). In the study group, 76% of patients were female. The largest patient group in 10-year intervals were 20–29 years old (22.5%) and 30–39 years old (20.0%) (Figure 2b).

### 2.2 Diagnostic test assays for specific IgE analysis

The RIDA qLine Allergy test system (R-Biopharm, Darmstadt, Germany) in manual processing tested 57 single-spotted, non-spiked allergens compromised on three different panels (*in vitro* diagnostic test panels A6143, A6243, A6343) as provided by the manufacturer and listed in Supplementary Table 1, for all serum samples. Immunoblot evaluation was carried out using an R-Biopharm AG validated 3-D flatbed scanner [RIDA qLine Scan (ZG1109)] in combination with the RIDA qLine Soft software (Z9995; Version 2.2.3). Automated ImmunoCAP Specific IgE tests (Thermo Fisher, Waltham, MA, USA) were conducted using the Phadia 200 instrument. They served as the primary reference method for all 57 allergens in all 200 human serum samples. The 3gAllergy Specific IgE Universal Kit (Siemens Healthcare, Forchheim, Germany) in conjunction with an IMMULITE 2000 Systems Analyzer was used as a secondary reference method to reanalyze samples with discrepant qualitative and quantitative results. Serum samples were stored at 2–8°C for the first week and then at −20°C, with consistent processing across all methods. Lipemic, hemolytic, icteric, or opaque samples, and those subjected to repeated freeze–thaw cycles, were excluded from the analysis. All test methods were conducted in accordance with the manufacturers’ instructions. Technical replicates were not included.

In the second part of this study, serum samples with CCD band positivity in the RIDA qLine Allergy results were preincubated with the RIDA CCD inhibitor (ZA0601) according to the manufacturer's instructions. The pretreated samples were then retested on the respective study panel using the manual RIDA qLine Allergy test system.

### 2.3 Statistics

**Figure 3:**
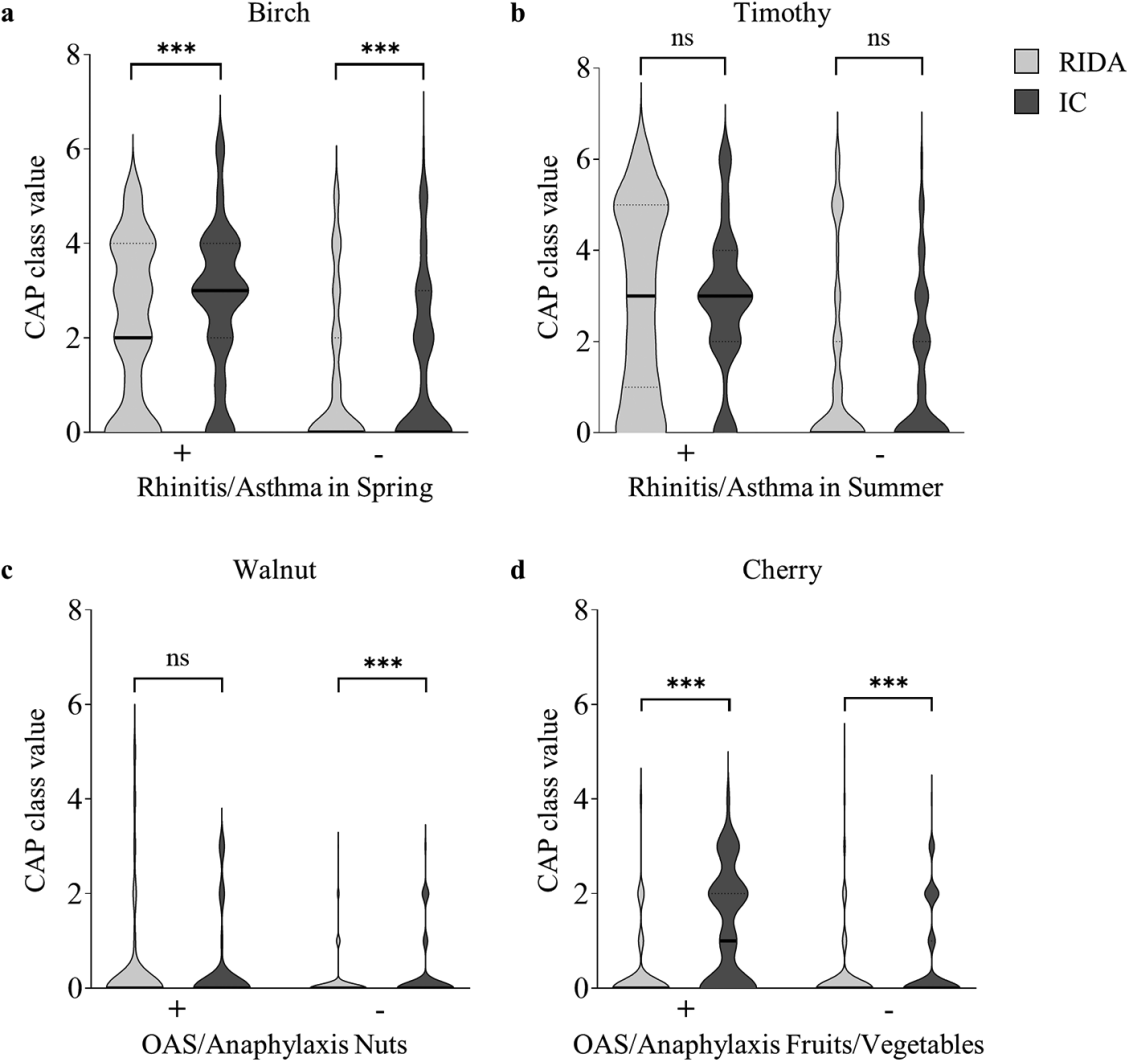
Comparative analysis of the clinical diagnoses and the corresponding sensitization profiles. Comparison of the positive (+) or negative (−) clinical diagnosis with the respective sensitization profile, shown as the CAP class value. CAP class values, as determined by the RIDA qLine Allergy test system (RIDA; light gray violin plot) or the ImmunoCAP Specific IgE test (IC; dark gray violin plot), were compared. The distribution of the CAP class values is depicted using violin plots, with the median represented by a solid line and the quartiles by dotted lines. Statistical analysis was performed using the Wilcoxon matched-pairs signed rank test to compare medians between groups (ns: not significant, ***p < 0.001). **(a)** Positive (*n* = 84) and negative (*n* = 116) clinical diagnoses of allergic rhinitis and/or allergic asthma in spring are compared to the CAP class values for the aeroallergen birch. **(b)** Positive (*n* = 65) and negative (*n* = 135) clinical diagnoses of allergic rhinitis and/or allergic asthma in summer are compared to the CAP class values for the aeroallergen timothy grass. **(c)** Positive (*n* = 41) and negative (*n* = 159) clinical diagnoses of oral allergy syndrome and/or anaphylaxis to nuts are compared to the CAP class values for the food allergen walnut. **(d)** Positive (*n* = 56) and negative (*n* = 144) clinical diagnoses of oral allergy syndrome and/or anaphylaxis to fruits/vegetables are compared to the CAP class values for the food allergen cherry.

**Figure 4:**
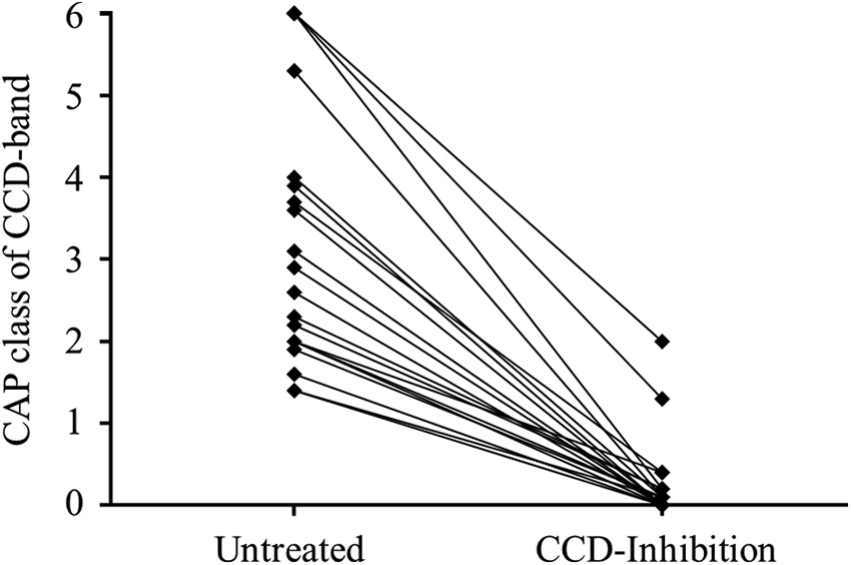
Clinical performance of the RIDA CCD inhibitor. Representative presentation of the CAP classes of the CCD band of the first randomly chosen 20 positive samples as detected with the RIDA qLine Allergy test system before (left, untreated) and after (right, CCD inhibition) incubation with the RIDA CCD inhibitor.

Data analysis was conducted using MedCalc Statistical Software (version 20.011). Quantitative data obtained from the CAP classes were reported as integers. Decimal values derived from the RIDA qLine Allergy test system were systematically rounded to the nearest lower integer. Concordance between the RIDA qLine Allergy and the ImmunoCAP and 3gAllergy platforms was assessed for binary outcomes (overall, positive, or negative percent agreement) and agreement was defined as the following: The difference (Δ) between the CAP classes of the obtained results (test vs. reference method) was ≤1. This comparison employed Cohen's kappa coefficient (*κ*) and provided 95% confidence intervals to quantify agreement levels. The interpretative framework for the kappa values adhered to the standard guidelines: *κ* <0 indicated poor agreement; 0–0.20 slight agreement; 0.21–0.40 fair agreement; 0.41–0.60 moderate agreement; 0.61–0.80 substantial agreement; and 0.81–1.00 represented almost perfect agreement. Statistical analysis and data visualization in Figures 2–4 were performed using GraphPad Prism 10.3.1.

## 3 Results

### 3.1 Agreement with the reference methods for aeroallergens and food allergens

The RIDA qLine Allergy results were compared to the initial testing results obtained with the ImmunoCAP system. The overall agreement for all 57 allergens was 91.8%, with a positive percent agreement of 61.6% and a negative percent agreement of 98.8% (Table 1). Retesting of discrepant results (difference >1 CAP class) was performed using the 3gAllergy system, which increased the overall agreement to 94.9%. The positive percent agreement improved to 75.1% and the negative percent agreement was 98.9%.

**Table 1:** Arithmetic mean and median inter-assay agreement.

	Initial testing with ImmunoCAP			Retesting with 3gAllergy		
	PPA (%)	NPA (%)	OA (%)	PPA (%)	NPA (%)	OA (%)
Arithmetic mean_all allergens_	61.6	98.8	91.8	75.1	98.9	94.9
Median_all allergens_	58.3	99.5	94.3	80.0	99.5	96.7
Median_25 percentile all allergens_	47.4	98.7	88.9	61.8	98.9	93.0
Median_75 percentile all allergens_	76.7	100	96.6	88.5	100	98.6

Arithmetic mean and median values for inter-assay agreement are represented for all 57 aero- and food allergens. The comparison involves the RIDA qLine Allergy test system compared with testing by ImmunoCAP Specific IgE test. Retesting of discrepant samples was done using the 3gAllergy Specific IgE Universal Kit. Positive percent agreement (PPA), negative percent agreement (NPA), and overall agreement (OA) compared to the results of the reference methods are shown.

### 3.2 Inter-assay agreement for aeroallergens

A comparison of the RIDA qLine Allergy results with ImmunoCAP results was performed as the initial testing for all aeroallergens and showed a substantial mean agreement for all aeroallergens (*κ* = 0.692) (Table 2). Among the inhalative allergens, the highest agreement was observed for meadow fescue (*κ* = 0.856), timothy (*κ* = 0.847), and orchard grass (*κ* = 0.827), while the lowest agreement was found for guinea pig (*κ* = 0.312), olive (*κ* = 0.521), and *Candida albicans* (*κ* = 0.529). The positive percent agreement ranged from 21.4% to 91.6%, while the negative percent agreement was no less than 88.8%. Retesting of discrepant results was performed using the 3gAllergy system. A comparative analysis between the outcomes of the retested samples and those obtained via the RIDA qLine Allergy demonstrated consistent or, in most cases, increased positive percent agreement for all retested samples, ranging from 40.0% to 100.0%. Negative percent agreement remained above 89.0% for all samples.

**Table 2:** Inter-assay agreement on aeroallergens.

					Initial testing with ImmunoCAP						Retesting with 3gAllergy			
	No. of tested sera	RIDA +	IC +	IC/3g +	PPA (%)	95% CI	NPA (%)	95% CI	*κ*		PPA (%)	95% CI	NPA (%)	95% CI
Meadow fescue	200	113	119	119	91.6	85.1–95.9	95.1	87.8–98.6	0.856		91.6	85.1–95.9	95.1	87.8–98.6
Timothy grass	200	109	120	117	89.2	82.2–94.1	97.5	91.3–99.7	0.847		91.5	84.8–95.8	97.6	91.6–99.7
Orchard grass	200	108	119	119	88.2	81.0–93.4	96.3	89.6–99.2	0.827		88.2	81.0–93.4	96.3	89.6–99.2
Cat	200	45	59	57	76.3	63.4–86.4	100.0	97.4–100.0	0.819		78.9	66.1–88.6	100.0	97.5–100.0
Rabbit	203	10	12	11	75.0	42.8–94.5	99.5	97.1–100.0	0.808		90.9	58.7–99.8	100.0	98.1–100.0
Birch	200	108	127	121	85.0	77.6–90.7	100.0	95.1–100.0	0.806		89.3	82.3–94.2	100.0	95.4–100.0
Ryegrass	200	101	117	117	84.6	76.8–90.6	97.6	91.6–99.7	0.800		84.6	76.8–90.6	97.6	91.6–99.7
Wheat	200	110	111	109	90.1	83.0–94.9	88.8	80.3–94.5	0.788		91.7	84.9–96.2	89.0	80.7–94.6
Mugwort	200	40	56	55	71.4	57.8–82.7	100.0	97.5–100.0	0.783		72.7	59.0–83.9	100.0	97.5–100.0
*Blomia tropicalis*	200	37	49	34	71.4	56.7–83.4	98.7	95.3–99.8	0.764		100.0	89.7–100.0	98.2	94.8–99.6
Rye	207	93	113	111	80.5	72.0–87.4	97.7	91.9–99.7	0.762		82.0	73.6–88.6	97.8	92.1–99.7
*Dermatophagoides pteronyssinus*	200	65	88	84	73.9	63.4–82.7	100.0	96.8–100.0	0.760		77.4	67.0–85.8	100.0	96.9–100.0
Horse	207	25	38	32	65.8	48.6–80.4	100.0	97.8–100.0	0.758		78.1	60.0–90.7	100.0	97.9–100.0
Amb., mugwort-l.	200	56	59	48	79.7	67.2–89.0	93.6	88.2–97.0	0.744		97.9	88.9–99.9	94.1	89.1–97.3
Alder	200	95	124	114	76.6	68.2–83.7	100.0	95.3–100.0	0.713		83.3	75.2–89.7	100.0	95.8–100.0
*Dermatophagoides farinae*	200	65	94	85	69.1	58.8–78.3	100.0	96.6–100.0	0.704		76.5	66.0–85.0	100.0	96.8–100.0
Oak	200	81	112	94	72.3	63.1–80.4	100.0	95.9–100.0	0.697		86.2	77.5–92.4	100.0	96.6–100.0
Hazel	200	91	124	97	73.4	64.7–80.9	100.0	95.3–100.0	0.677		89.7	81.9–94.9	96.1	90.4–98.9
*Penicillium chrysogenum/chrysogenum*	205	9	12	10	58.3	27.7–84.8	99.0	96.3–99.9	0.649		80.0	44.4–97.5	99.5	97.2–100.0
Dog	200	29	53	36	54.7	40.5–68.4	100.0	97.5–100.0	0.640		80.6	64.0–91.8	100.0	97.8–100.0
*C. herbarum*	212	7	16	13	43.8	19.8–70.1	100.0	98.1–100.0	0.590		53.8	25.1–80.8	100.0	98.2–100.0
Cockroach	209	22	43	26	48.8	33.3–64.5	99.4	96.7–100.0	0.589		80.8	60.6–93.4	99.5	97.0–100.0
*Alternaria alternata/ tenuis*	213	15	32	30	43.8	26.4–62.3	99.4	97.0–100.0	0.553		46.7	28.3–65.7	99.5	97.0–100.0
Ribwort plantain	200	30	64	37	46.9	34.3–59.8	100.0	97.3–100.0	0.545		81.1	64.8–92.0	100.0	97.8–100.0
*Aspergillus fumigatus*	212	9	19	13	42.1	20.3–66.5	99.5	97.1–100.0	0.545		61.5	31.6–86.1	99.5	97.2–100.0
*C. albicans*	208	14	28	17	42.9	24.5–62.8	98.9	96.0–99.9	0.529		70.6	44.0–89.7	99.0	96.3–99.9
Olive	200	38	80	73	47.5	36.2–59.0	100.0	97.0–100.0	0.521		52.1	40.0–63.9	100.0	97.1–100.0
Guinea pig	202	4	14	10	21.4	4.7–50.8	99.5	97.1–100.0	0.312		40.0	12.2–73.8	100.0	98.1–100.0
Arithmetic mean_Aeroallergens_	—	54.61	71.50	63.89	66.58		98.59		0.692		78.49		98.53	

Overview of inter-assay agreement between the RIDA qLine Allergy test system and ImmunoCAP Specific IgE test for IgE of all 28 aeroallergens. Retesting of all discrepant results was done with the 3gAllergy Specific IgE Universal Kit. Listed are the number of sera tested and the number of sera tested with positive results (CAP class ≥1) for each allergen as determined by RIDA qLine Allergy (RIDA), ImmunoCAP (IC), or 3gAllergy (3 g). The positive percent (PPA) and negative percent (NPA) agreement with the corresponding 95% confidence interval (95% CI), and Cohen’s kappa (*κ*) are given. *κ* < 0 indicates poor agreement (red); 0–0.20 slight agreement (orange); 0.21–0.40 fair agreement (yellow); 0.41–0.60 moderate agreement (light green); 0.61–0.80 substantial agreement (green); and 0.81–1.00 represents almost perfect agreement (dark green).

### 3.3 Inter-assay agreement for food allergens

A comparison of the initial testing of all food allergens using ImmunoCAP with RIDA qLine Allergy showed a substantial mean agreement for all food allergens (*κ* = 0.626) (Table 3). The highest agreement among the food allergens was observed for sardine (*κ* = 1.000), casein (*κ* = 0.960), and cod (*κ* = 0.957), while the lowest agreement was found for hazelnut (*κ* = 0.082), shrimp (*κ* = 0.366), and apple (*κ* = 0.442). The arithmetic mean of the number of positive samples for any food allergen with CAP ≥1 was 20.03 for the RIDA qLine Allergy results, and 39.72 for the ImmunoCAP results (30.24 for 3gAllergy results). In contrast, the arithmetic mean of positive samples was higher for all aeroallergens: 54.61 for RIDA qLine Allergy, 71.50 for ImmunoCAP, and 63.89 for 3gAllergy (Table 2). Excluding hazelnut, which is commonly augmented/spiked with molecular allergens in ImmunoCAP, the positive percent agreement for food allergens ranged from 27.1% to 100.0%. Only retesting discrepant results using the 3gAllergy kit and comparing them with the RIDA qLine Allergy results revealed a consistent or increased positive percent agreement for all samples ranging from 35.0% to 100.0% (hazelnut excluded). The negative percent agreement with the RIDA qLine Allergy results varied from 92.8% to 100.0% compared to ImmunoCAP, or from 94.0% to 100.0% compared to the ImmunoCAP results retested with 3gAllergy.

**Table 3:** Inter-assay agreement: food allergens.

					Initial testing ImmunoCAP						Retesting 3gAllergy			
	No. of tested sera	RIDA +	IC +	IC/3g +	PPA (%)	95% CI	NPA (%)	95% CI	*κ*		PPA (%)	95% CI	NPA (%)	95% CI
Sardine	206	7	7	7	100.0	59.0–100.0	100.0	98.2–100.0	1.000		100.0	59.0–100.0	100.0	98.2–100.0
Casein	212	13	14	13	92.9	66.1–99.8	100.0	98.2–100.0	0.960		100.0	75.3–100.0	100.0	98.2–100.0
Cod	211	13	12	12	100.0	73.5–100.0	99.5	97.2–100.0	0.957		100.0	73.5–100.0	99.5	97.2–100.0
Egg yolk	212	10	12	12	83.3	51.6–97.9	100.0	98.2–100.0	0.904		83.3	51.6–97.9	100.0	98.2–100.0
Chicken	212	10	13	11	76.9	46.2–95.0	100.0	98.2–100.0	0.862		90.9	58.7–99.8	100.0	98.2–100.0
Milk	214	14	22	16	63.6	40.7–82.8	100.0	98.1–100.0	0.758		87.5	61.7–98.4	100.0	98.2–100.0
Crab	207	20	29	19	65.5	45.7–82.1	99.4	96.9–100.0	0.747		100.0	82.4–100.0	99.5	97.1–100.0
Cashew nut	210	22	13	12	100.0	75.3–100.0	95.4	91.5–97.9	0.721		100.0	73.5–100.0	94.9	90.9–97.6
Buckwheat flour	206	24	40	31	60.0	43.3–75.1	100.0	97.8–100.0	0.707		77.4	58.9–90.4	100.0	97.9–100.0
Sesame	200	24	42	30	57.1	41.0–72.3	100.0	97.7–100.0	0.678		80.0	61.4–92.3	100.0	97.9–100.0
Egg white	217	13	25	21	52.0	31.3–72.2	100.0	98.1–100.0	0.657		61.9	38.4–81.9	100.0	98.1–100.0
Mackerel	205	6	9	9	55.6	21.2–86.3	99.5	97.2–100.0	0.655		55.6	21.2–86.3	99.5	97.2–100.0
Tomato	200	32	47	44	59.6	44.3–73.6	97.4	93.4–99.3	0.640		70.5	54.8–83.2	99.4	96.5–100.0
Almond	200	26	44	40	54.5	38.8–69.6	98.7	95.4–99.8	0.624		60.0	43.3–75.1	98.8	95.6–99.8
Tuna	210	7	12	9	50.0	21.1–78.9	99.5	97.2–100.0	0.615		66.7	29.9–92.5	99.5	97.3–100.0
Wheat flour	200	21	42	26	50.0	34.2–65.8	100.0	97.7–100.0	0.612		80.8	60.6–93.4	100.0	97.9–100.0
Rye flour	207	26	53	33	49.1	35.1–63.2	100.0	97.6–100.0	0.589		72.7	54.5–86.7	98.9	95.9–99.9
Orange	209	23	45	40	48.9	33.7–64.2	99.4	96.7–100.0	0.587		55.0	38.5–70.7	99.4	96.7–100.0
Walnut	200	20	34	20	50.0	32.4–67.6	98.2	94.8–99.6	0.576		85.0	62.1–96.8	98.3	95.2–99.7
Peanut	200	32	65	41	47.7	35.1–60.5	99.3	95.9–100.0	0.541		75.6	59.7–87.6	99.4	96.5–100.0
Strawberry	208	24	52	31	44.2	30.5–58.7	99.4	96.5–100.0	0.531		71.0	52.0–85.8	98.9	96.0–99.9
Celery	200	48	75	50	52.0	40.2–63.7	92.8	86.8–96.7	0.483		78.0	64.0–88.5	94.0	88.9–97.2
Soya bean	207	19	43	31	39.5	25.0–55.6	98.8	95.7–99.9	0.483		54.8	36.0–72.7	98.9	96.0–99.9
Cherry	200	33	79	73	41.8	30.8–53.4	100.0	97.0–100.0	0.465		45.2	33.5–57.3	100.0	97.1–100.0
Carrot	200	30	68	52	41.2	29.4–53.8	98.5	94.6–99.8	0.459		55.8	41.3–69.5	99.3	96.3–100.0
Clam	205	9	20	20	35.0	15.4–59.2	98.9	96.2–99.9	0.449		35.0	15.4–59.2	98.9	96.2–99.9
Apple	200	31	72	74	40.3	28.9–52.5	98.4	94.5–99.8	0.442		41.9	30.5–53.9	100.0	97.1–100.0
Shrimp	215	13	48	15	27.1	15.3–41.8	100.0	97.8–100.0	0.366		86.7	59.5–98.3	100.0	98.2–100.0
Hazelnut	200	11	115	85	9.6	4.9–16.5	100.0	95.8–100.0	0.082		12.9	6.6–22.0	100.0	96.8–100.0
Arithmetic mean_Foodallergens_	—	20.03	39.72	30.24	56.81		99.07		0.626		71.87		99.21	

Overview of inter-assay agreement between the RIDA qLine Allergy test system and ImmunoCAP Specific IgE test for IgE of all 29 food allergens. Retesting of all discrepant results was done with the 3gAllergy Specific IgE Universal Kit. Listed are the number of sera tested and the number of sera with positive results (CAP class ≥1) for each allergen as determined by RIDA qLine Allergy (RIDA), ImmunoCAP (IC), or 3gAllergy (3 g). The positive percent (PPA) and negative percent (NPA) agreement with the corresponding 95% confidence interval (95% CI), and Cohen’s kappa (*κ*) are given. *κ* < 0 indicates poor agreement (red); 0–0.20 slight agreement (orange); 0.21–0.40 fair agreement (yellow); 0.41–0.60 moderate agreement (light green); 0.61–0.80 substantial agreement (green); and 0.81–1.00 represents almost perfect agreement (dark green).

### 3.4 Inter-assay agreement with clinical diagnosis

The presumed allergological diagnoses of the individual patients were compared with allergen-specific IgE test outcomes obtained using the RIDA qLine Allergy and ImmunoCAP systems (Figures 3a–d). For all patients diagnosed with allergic rhinitis and/or allergic asthma in spring, the median CAP class value for the regionally most relevant spring aeroallergen, birch pollen, was 2 using RIDA qLine Allergy and significantly higher at 3 using ImmunoCAP (Figure 3a). The CAP class values for patients with a negative diagnosis showed a clear downward trend, with a median of 0 for both systems, while the values obtained with RIDA qLine Allergy remained significantly lower than those of the ImmunoCAP system. Comparison of the CAP class values for the aeroallergen timothy grass pollen in patients with a positive diagnosis of allergic rhinitis and/or allergic asthma in summer revealed an agreement at a median of 3 for both test systems, while a distinct reduction to a median of 0 was observed in patients with an absent diagnosis (Figure 3b). The test results for the food allergen walnut showed a comparable median CAP class value at 0 for patients with a positive diagnosis of OAS and/or anaphylaxis to nuts. For patients with a negative diagnosis, the CAP class values showed a slight decrease in the measurements obtained using RIDA qLine Allergy, with an even more modest decrease observed in ImmunoCAP (Figure 3c). In patients with a positive diagnosis of OAS and/or anaphylaxis to fruits/vegetables, specific IgE to cherry showed a median CAP class of 1 when measured by ImmunoCAP, whereas lower values were observed in patients without this diagnosis (Figure 3d). In contrast, the results obtained by RIDA qLine Allergy were significantly lower, with a median of 0 in both categories.

### 3.5 CCD inhibition analysis

The first randomly chosen 20 patient samples that tested positive for a CCD band were subsequently incubated with the RIDA CCD inhibitor and then retested for their CCD band CAP class. All patient samples exhibited a significant decrease in CAP class (Figure 4). Except for two patient samples, all inhibitor-treated samples retested negative. The two affected samples that still showed positivity of the CCD band were initially in the highest CAP class before treatment, which led to a distinct drop in the respective value after CCD inhibition without total clearance.

## 4 Discussion

The comparative analysis of serum IgE levels, measured by RIDA qLine Allergy and the designated monoplex gold standard ImmunoCAP, exhibited a high mean overall concordance of 91.8% across all tested allergens. This concordance was further enhanced to 94.9% following retesting of inconsistent results using the 3gAllergy system. For both aeroallergens and food allergens, the inter-assay agreement was substantial, with an average Cohen's kappa coefficient exceeding 0.61. The agreement observed between the two test methods aligns with findings from previous studies comparing ImmunoCAP with other multiplex immunoblot-based systems (17–19). In the detailed evaluation of sIgE for aeroallergens and food allergens, several allergens demonstrated lower concordance, with *κ* ≤0.61, indicating moderate to fair agreement. Among these, certain allergens were characterized by a low incidence of positivity, with guinea pig, *Aspergillus fumigatus*, *Cladosporium herbarum,* and the food allergen clam each yielding ≤20 positive results as tested via initial testing with the ImmunoCAP. Additional allergens, including aeroallergens such as ribwort plantain and cockroach and food allergens such as shrimp and walnut, exhibited a greater positive concordance exceeding 30% upon retesting discrepant results utilizing the 3gAllergy system. Discrepancies in sIgE values, particularly in cut-off values across different testing systems, have also been highlighted in several previous studies and were attributed to technical differences among the applied tests (20–22).

Initial testing using ImmunoCAP revealed a notably low positive percent agreement with the RIDA qLine Allergy result of 9.6% in the detection of sIgE for hazelnut, which only slightly increased to 12.9% upon retesting. To enhance the analytical sensitivity for detecting hazelnut-specific IgE, ImmunoCAP incorporated molecular hazelnut allergens into their hazelnut extract, a process termed “spiking” (23). This modification involved the addition of the PR-10 protein Cor a 1, which is known for its cross-reactivity with birch pollen but thereby also resulting in a comparatively enhanced IgE detection capacity (24). These technical adjustments of the extract provide a hint that the laborious development of an optimized allergen extract may at least be equally important as the test method employed. A prospective approach is the use of molecular components instead of traditional allergen extracts in immunoblot assays as this enhances comparability by ensuring consistent measurement of allergenic proteins (18, 21).

For the inhalative allergens birch and timothy grass pollen, for which sensitization is most common in the German population, assessing the correlation of CAP classes as measured by RIDA qLine Allergy or ImmunoCAP has demonstrated a consistent pattern of elevated CAP classes in conjunction with positive clinical diagnoses and are lower in cases of absent diagnoses (1). This observation corresponds with the seasonal appearances of birch tree pollen in spring and timothy grass pollen in summer, highlighting an expected concordance of sensitization with probable clinical relevance for both allergens (25, 26).

Sensitization to the food allergens walnut and cherry is also commonly observed, both of which are associated with pollen-related cross-reactivity, also known as OAS (1, 27, 28). Walnut shows higher CAP class values for both test systems for patients with a known OAS or anaphylaxis to nuts, which drops in particular with RIDA qLine Allergy in the case of a negative diagnosis. This contrasts with the continuously low CAP class values measured with RIDA qLine Allergy for the allergen cherry independent of the clinical diagnosis, while ImmunoCAP displayed an increased CAP class value in patients with an assigned OAS and/or anaphylaxis to food allergens. It should be noted that in this study there was no explicit but only a general categorization into the clinical categories OAS and/or anaphylaxis to nuts or fruits and vegetables. However, deficiencies in allergenic components within natural extracts due to manufacturing problems are commonly observed in cherry and further food allergens with serological cross-reactivity to pollen, resulting in the development of recombinant allergens to improve the sensitivity of sIgE detection (28–30).

In this study, ImmunoCAP was selected as the primary reference method due to its widespread recognition as a standard method for monoplex IgE measurement and its demonstrated level of agreement with skin prick tests (31–33). Correspondingly, this study did not evaluate the correlation between RIDA qLine Allergy results and skin prick tests, nor did it assess the alignment of the findings with clinical diagnoses of the patients established following comprehensive allergy diagnostics. In the context of *in vitro* IgE measurements, it is important to consider that lower levels of sIgE indicate a reduced risk for clinically relevant sensitization, but are also linked to increased inter-assay discrepancies (34, 35).

To assess the efficacy of the RIDA CCD inhibitor, this study involved incubation of samples that initially exhibited positivity to CCDs with the CCD inhibitor; subsequent testing showed a successful reduction of the CCD bands to below detectable levels in all but two instances. These two exceptions, which initially displayed the highest CCD-CAP class values of 6, imply that adjusting the inhibitor concentration may be necessary to achieve sufficient reduction in cases of high initial sIgE levels to CCDs. While this study did not retest treated samples to evaluate the effect of CCD inhibition on reducing false-positive *in vitro* results, previous research has demonstrated that implementing CCD inhibition successfully enhances the sensitivity for detecting relevant IgE interactions (8, 9). Furthermore, even with the prospective use of recombinant allergens in immunoblot assays, CCD inhibitors remain relevant to further mitigate interference with cross-reactive carbohydrate determinants (36).

## 5 Conclusion

This study presents a comparative analysis of the RIDA qLine Allergy test system with the ImmunoCAP Specific IgE test, and the 3gAllergy Specific IgE Universal Kit, considering the correlation with the clinical presentations observed in the analyzed patients. The results highlight a concordance observed in serum sIgE levels measured by RIDA qLine Allergy and ImmunoCAP, exhibiting a high mean overall agreement of 91.8% across various allergens, with a greater overall agreement of 94.9% upon retesting discrepant results with 3gAllergy. These results also emphasize that sIgE values are not universally interchangeable and require careful evaluation of potential confounding factors and highlight the efficacy of diverse diagnostic technologies in determining sIgE serum levels. The study further suggests that multiplex screening tools, such as RIDA qLine Allergy, could be particularly effective as a first-line “bottom-up” diagnostic approach. Their advantages include cost-effectiveness, reduced need for specialized equipment and training, and the ability to provide semi-quantitative analyses, making them particularly valuable in resource-limited settings. Broad sIgE measurements are particularly valuable in the diagnostic assessment of polysensitized patients, yet they must be interpreted within the clinical context and relevance to ensure their applicability in allergy diagnostics.

## Data Availability

The raw data supporting the conclusions of this article will be made available by the authors, without undue reservation.
