# Supplementary material for: Comparison of the new RIDA qLine Allergy multiparameter immunoblot and the ImmunoCAP Specific IgE test for the identification of clinically relevant food and aeroallergen allergies
Source: Front Allergy. 2025 Jan 10;5:1496882. doi: 10.3389/falgy.2024.1496882 (PMC11757282; doi:10.3389/falgy.2024.1496882)
Supplement: Supplementary file 1 [file Table1.pdf]

Supplementary Material

Supplementary Table 1

Table of Study Panel I-III of RIDA qLine Allergy Test System.
Overview of the 57 aeroallergens or food allergens investigated in this study, categorized in distinct study panels. Each study panel compromises 20 allergens (17 allergens for study panel III).

Panel I	Panel II	Panel III
D. pteronyssinus	Hazelnut	Cockroach
D. farinae	Peanut	Cashew nut
Alder	Walnut	Shrimp
Birch	Almond	Mackerel
Hazel	Milk	Sardine
Oak	Egg white	Tuna
Olive	Egg yolk	Clam
Rye	Casein	Buckwheat flour
Mugwort	Chicken	Strawberry
Ribwort plantain	Celery	Cherry
Cat	Carrot	Wheat
Horse	Tomato	Meadow fescue
Dog	Cod	Orchard grass
Guinea pig	Crab	Ryegrass
Rabbit	Orange	Timothy
Amb., mugwort-leaved	Apple	B. tropicalis
P. notatum/chrysogenum	Wheat flour	C. albicans
C. herbarum	Rye flour	
Aspergillus fumigatus	Sesame	
A. alternata/tenuis	Soya bean	
